# Tunable Work Functions in Plasmonic Metals

**DOI:** 10.3390/nano15191483

**Published:** 2025-09-29

**Authors:** Kanij Mehtanin Khabir, Leila Hesami, Anthony P. Martin, Jawuan Wilson, Chi Yang, Mikhail A. Noginov

**Affiliations:** 1 Center for Materials Research, Norfolk State University, Norfolk, VA 23504, USA; l.hesami@spartans.nsu.edu (L.H.); cyang@nsu.edu (C.Y.); mnoginov@nsu.edu (M.A.N.); 2 Engineering Department, Virginia State University, Petersburg, VA 23806, USA; amar4357@students.vsu.edu; 3 Department of Science, Mathematics, Computer Science and Engineering Technology, Elizabeth City State University, Elizabeth City, NC 27909, USA; jawilson658@students.ecsu.edu

**Keywords:** work function, BITh, kelvin probe, quartz lamp, photopolymerization

## Abstract

We have studied the effect of BITh molecules on the work functions of Ag and Au, with and without quartz lamp illumination. Silver and gold films coated with BITh molecules were fabricated and studied in reflection and Kelvin Probe experiments. The deposition of BITh films on Ag and Au reduced their work functions (in agreement with our recent study, wherein a similar reduction was caused by the deposition of a PMMA polymer). Illumination of the BITh-coated samples with a quartz lamp caused reductions in work functions by several tens of meV, which (almost) returned to their original values when the light was turned off. The characteristic time of this process (~15 min) was much shorter than that of photopolymerization (~180 min), suggesting that these two phenomena are nearly independent of each other. The effects of the Au substrates were qualitatively similar to those of the Ag substrates. Our findings pave the way to fundamental studies and applications of light–matter interactions.

## 1. Introduction

Work function (WF) is a fundamental property of metals [1,2,3,4] that significantly influences their performance in applications ranging from electronic devices [5] and photovoltaic systems [6] to emerging nanophotonic systems [7,8]. In this study, we explore how photo-exposure of BITh molecules affects the work functions of Ag and Au substrates [9,10].

In our WF study [7], the samples were single and multi-layered films of Rh590:PMMA [Rh590 = Rhodamine 590 laser dye and PMMA = Poly methyl methacrylate] and Au films deposited on plain glass. Unlike our previous studies [8,11,12,13] that treated WF as a purely surface related property, we observed that the WF of a metallic surface can be influenced by a dielectric material located 10 to 100 nm away from the metal surface. In particular, we have found that glass substrates reduce the work functions of Au surfaces separated from glass by ≈50 nm of gold. In contrast, a dye-doped polymer Rh590:PMMA increases the WF when an Au film is deposited on top of it but decreases the WF when (Rh590:PMMA) is deposited on top of Au. This behavior is consistent with electron transfer from dye-doped polymeric films to metals [7,13]. Additionally, the WFs of Metal–Insulator–Metal (MIM) waveguides and Fabry–Perot cavities were nearly identical to those of thick gold films.

A simple model [13] offers a qualitative explanation for these findings by considering how charged molecules adhere to a metal surface. This interaction creates a double layer of charge, which influences the energy barrier for electrons escaping the metal either enhancing or hindering their motion and as a result, modifies the work function by lowering or raising it.

**Figure 1:**
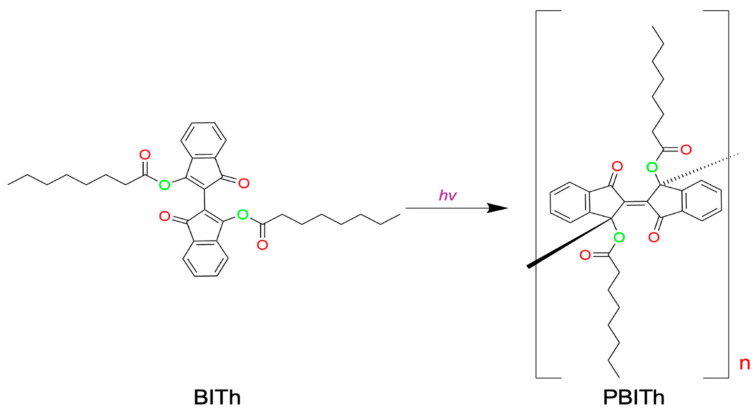
Schematic demonstration of the photopolymerization of BITh [14].

**Figure 2:**
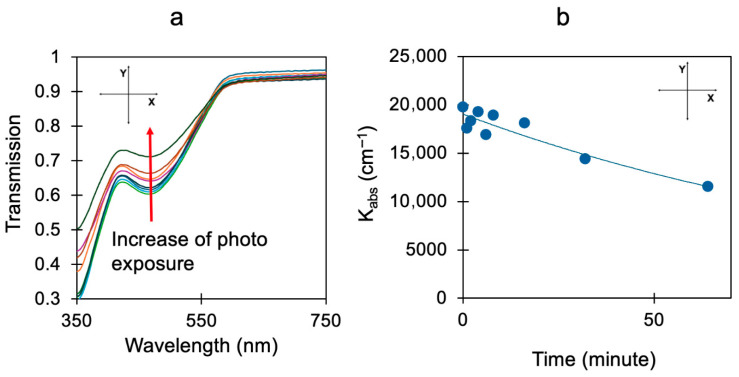
(**a**) Transmission spectrumof BITh, showing the decreases in the width of the 480 nm absorption band with increasing photo-exposure and photopolymerization; adapted from Ref. [14]. (**b**) Kinetics of the reduction in the 480 nm absorption band, corresponding to the BITh monomer --> polymer transformation; adapted from Ref. [14].

In our recent work [14], we studied how metal–dielectric substrates have effects on the photopolymerization of BITh molecules (BITh = [2,2′-bi-1H-indene]-1,1′-dione-3,3′-diyldiheptanoatecarboxylate monomer), as shown in Figure 1. After synthesizing the BITh monomer, we coated it onto different varieties of dielectric, metallic, and metal–dielectric substrates [15,16]. These coated films were then exposed to UV–Visible radiation from a Xe lamp, which initiated the photo-polymerization of monomer molecules. We tracked the polymerization rate by observing the decrease in the ~480 nm absorption peak presented in the monomer but absent in the polymer over time, as illustrated in Figure 2a,b.

We found that the rate of photopolymerization was relatively high if the monomer film was deposited on top of silver separated from the monomer by a thin insulating MgF_2_ layer preventing a charge transfer [14]. At the same time, the rate of photopolymerization became threefold larger if the monomer was deposited on the Ag film directly and charge transfer was allowed.

At this time, we report on control of the work functions of BITh-coated Ag and Au films with external light.

## 2. Sample Fabrication

**Figure 3:**
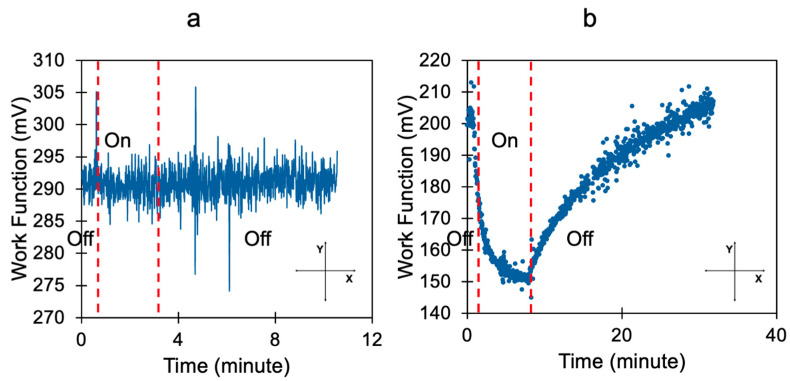
Work functions of (**a**) pristine and (**b**) BITh-coated Ag films measured with the light turned on and off. In both measurements, light was off at the initial moment of time t = 0. One can see that the WF in the coated sample is significantly smaller than that in the pristine Ag sample. One can also see the reduction in WF upon photoillumination.

**Figure 4:**
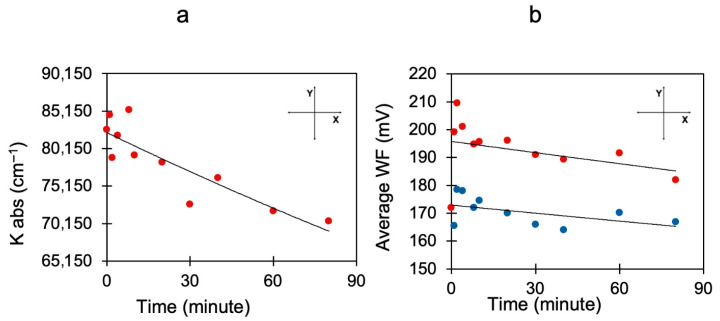
BITh on Ag. (**a**) Kinetics of the monomer → polymer transformation obtained from reflection spectra taken at different illumination times (fluences). (**b**) Work function kinetics measured when the light was turned on and off. The work functions were measured two times, before photo-exposure and immediately after photo-exposure. Blue circles: Work functions measured immediately after photo-exposure. Red circles: work functions measured before photo-exposure.

**Figure 5:**
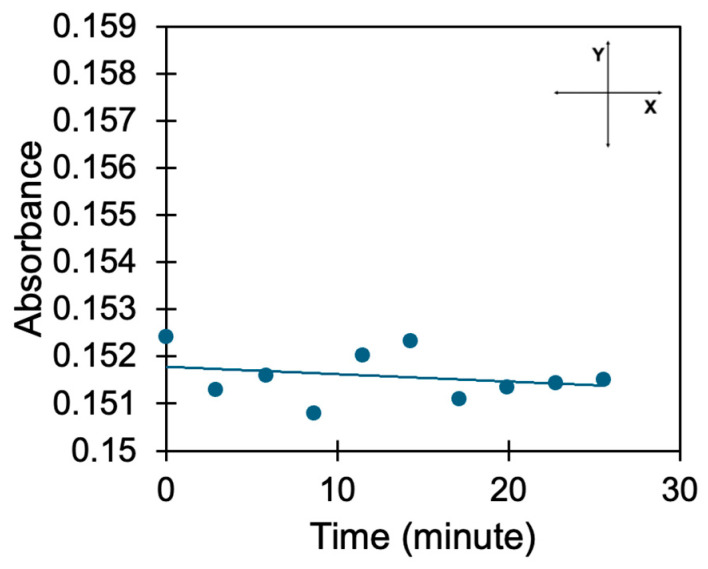
Absorbance at 480 nm measured back-to-back with the light turned off after initial illumination.

**Figure 6:**
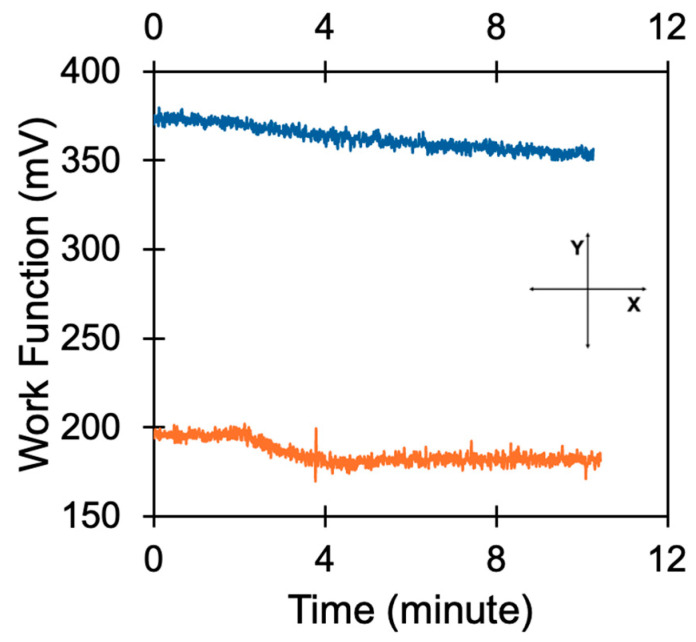
Work function of pristine Au (blue) and Au coated with BITh (orange) film with the light turned on and off.

The BITh monomer was synthesized using the method described in Refs. [14,17,18]. In brief, ~130 nm thin Ag and Au films were deposited using a thermal vapor deposition machine (Nano 36 from J. Kurt Lesker, Jefferson Hills, PA, USA). In most measurements, BITh films were deposited using a Spin Coater (from Specialty Coating System), as shown in Figure 3b, Figure 4, and Figure 5. This enabled us to produce uniform, well-controlled thin films suitable for reliable Kelvin Probe and reflection measurements. At the same time, in some particular experiments, BITh was deposited via drop casting, Figure 6, where variations in film morphology were intentionally explored. The choice of deposition method was guided by the specific objectives of each measurement, recognizing that film morphology and molecular orientation both influenced by deposition technique may significantly affect the interfacial electronic structure and measured work function. The film thickness was measured with a stylus profilometer (Dek-Tak XT from Bruker, Billerica, MA, USA).

## 3. Work Functions of Silver Substrates

In the first set of experiments, our samples were ~130 nm thin Ag films placed above or below the layer of BITh molecules. We found that in agreement with Ref. [7], the deposition of organic material (~27 nm) on top of the Ag film reduces the work functions by several tens of hundred electron volts, compare to the work functions in Figure 3a,b measured at t—0 without light illumination.

The measurements were performed using the Kelvin Probe (KP) apparatus from KP Technologies [11,12]. The tip of the Kelvin Probe is made of gold alloy. The tip sample distance was kept at approximately 0.2–2.0 mm [19]. All experiments were conducted under ambient conditions (room temperature ~20–25 °C and relative humidity ~45%). Vibration effects were minimized using an anti-vibration optical table. These conditions were carefully maintained to ensure the precision and reliability of the work function data.

In the next particular experiment, the BITh/Ag samples were illuminated, on and off, with a quartz lamp, which was a part of the Kelvin Probe apparatus. Their work functions and the reflection spectra (used to determine the kinetics of photopolymerization) were studied, as functions of time, using the Kelvin Probe and the spectrophotometer Lambda 900 from Perkin Elmer (Waltham, MA, USA).

We found that in BITh-coated Ag films, the work function decreased by several hundred milli-electron-volts during photo-exposure and almost (although not exactly) returned to its original value when the light was turned off, Figure 3b. The characteristic time scale of the cycle was ~15 min, strongly different from the characteristic time of photopolymerization, measured in the K_abs_ vs. photo-exposure time experiment, Figure 4a.

In the next particular experiment, the work functions were measured two times, before photo-exposure (red circles in Figure 4b), and immediately after photo-exposure (blue circles in Figure 4b), resulting in the kinetics consisting of two sets of experimental points. This is further evidence of the reduction in the WF upon illumination.

To determine whether BITh polymer returns back to the monomer state in the absence of light illumination, we photo-exposed the samples for ~10 min and then measured ten “back-to-back” reflection spectra with the excitation light turned off (each spectral scan was ~3 min long). We did not see any growth of the 480 nm absorption band when the light was off, which was evidence of the lack of reversibility of photopolymerization with no light, Figure 5. The corresponding kinetics of the “absorbance vs. time” experiment was nearly flat, with the characteristic decay time equal to 1773 s (29 min). This proves the lack of a direct relationship between WF and photopolymerization.

Note that, the work function did not change at photo-exposure of pristine silver films as well as Ag films coated with the PMMA polymer.

## 4. Work Functions of Gold Substrates

Qualitatively similar, although weaker, effects have been observed in Au based samples. (i) In pristine Au, the WF (measured relative to the tip) changes from ≈ 375 meV (at t = 0), Figure 6, to ≈350 meV (at t ≈ 10 min). (ii) BITh coated on Au changes the WF values from ≈ 196 meV (at t = 0) to ≈180 meV (at t≈10 min), Figure 6. (iii) Illumination of BITh on Au with the quartz lamp further reduces the work function of Au to ≈170 meV (over 3 min). However, recovery of the WF to its original value with the light turned off is much longer than that on top of the Ag substrate. (iv) Similarly to the case of Ag substrates, the effect of photo-illumination on the WF was specific to BITh, and no similar effect was observed in the Au film coated with PMMA.

## 5. Discussion

Three key processes described in this work, (i) photopolymerization of BITh, (ii) the effect of BITh on WFs of Ag and Au without illumination, and (iii) the effect of light illumination on the WFs of BITh-coated Ag and Au, are briefly discussed below.

### 5.1. Photopolymerization

**Figure 7:**
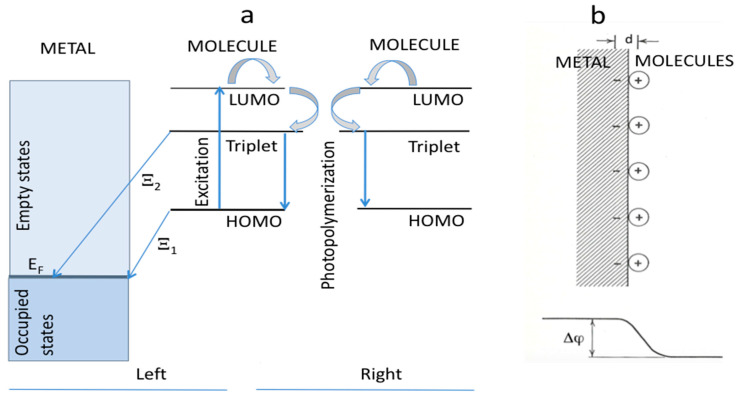
(**a**) Right: schematics of photopolymerization. Left: schematics of molecule → metal charge transfer. (**b**) Double layer of charges [13].

The polymerization mechanism of BITh is thought to follow a pathway analogous to that observed in diene compounds [14,17,20,21]. As reported in Refs. [14,17], the spectral band of BITh near 480 nm arises from π electron delocalization (π → π*) and intramolecular donor acceptor interactions (n → π*). Upon light absorption, the monomer reaches a singlet excited state (S_1_), which exhibits mixed ππ* and nπ* characters with intramolecular charge transfer and a relatively long lifetime. This S_1_ energy can undergo intermolecular transfer or transition via intersystem crossing to the lower energy triplet state (T_1_), as illustrated in Figure 7a (right). The T_1_ state of BITh forms a localized biradical that couples with lattice phonons [14,17,22,23], leading to exciton phonon interactions. This coupling induces lattice distortion, creating a trap that facilitates photoreaction initiation with adjacent T_1_ excited monomers (Figure 7a (right); see Refs. [14,17,22,23,24,25] for details). While previous studies [17,25] have suggested the possibility of polymer → monomer depolymerization, our experimental observations did not support the occurrence of this process.

### 5.2. Effect of BITh on the Work Functions of Ag and Au

Work function measurements are highly sensitive to the condition of the surface, especially in the presence of an adsorbed surface layer of foreign atoms [11]. When an atom approaches a metal surface, and it possesses an electron that can be more stable within the metal than in the atom itself, the electron may transfer to the metal, leaving behind a positively charged ion. This charge transfer mechanism, illustrated in Figure 7a (left), is referred to here as process Ξ_1_. Electrostatic attraction occurs between the negatively charged metal surface and the resulting positively charged ion, causing the ion to adhere to the surface (Figure 7b).

If these ions form a complete monoatomic layer on the metal, they create a double layer of charges, as shown in Figure 7b. This configuration generates an electric field between the two layers (‘d’ means the distance between two charge sheets here). As a result, extracting an electron from the metal requires less energy, since the internal electric field within the surface layer assists the process. Consequently, a monoatomic layer of electropositive atoms effectively lowers the surface’s work function [13].

### 5.3. Effect of Light Illumination

If the lifetime and excited state population of the triplet state T_1_ are high and the rate of the charge transfer Ξ_2_ originating from the triplet state is high as well, then light illumination of the BITh/metal sample can affect the charge of the double layered sheet and the effective work function of the metal coated with BITh. Depending on the multiple system parameters, including the absorption coefficient of BITh, pumping intensity, lifetime of the triplet state, density of states in metal, and charge transfer rates Ξ_1_ and Ξ_2_, the change in the work function under light illumination can be positive or negative. This is the subject of a future study to be published elsewhere.

## 6. Summary

To summarize, we have studied the effect of BITh molecules on the work functions of Ag and Au, with and without quartz lamp illumination. Silver and gold films coated with BITh molecules were fabricated and studied in reflection and Kelvin Probe experiments. BITh was chosen for this study due to its well-known photopolymerization behavior and our prior experience with the material. Its strong absorption near 480 nm allows for clear tracking of polymerization under illumination. This makes it a suitable candidate for investigating how different substrate types, especially metallic and metal dielectric combinations, influence both polymerization dynamics and work function changes. We have found that deposition of BITh films on Ag and Au reduces their work functions (in agreement with Ref. [7], wherein a similar reduction was caused by the deposition of an undoped or dye-doped PMMA polymer). Illumination of the BITh-coated samples caused a reduction in work functions (by several tens of meVs), which (almost) returned to their original values when the light was turned off. The characteristic time of this process (~15 min) was much shorter than the time of photopolymerization (~180 min), suggesting that these two phenomena are nearly independent of each other. The effects of the Au substrates were qualitatively similar to those of the Ag substrates. The mechanisms of photopolymerization and tunable work function are the subjects of a further study to be published elsewhere. The demonstrated light-induced control of the work functions paves the way for applications in active metamaterials, plasmonics, and nanophotonics.

## Data Availability

The original contributions presented in this study are included in the article. Further inquiries can be directed to the corresponding author.
